# Evaluating adipose‐derived stem cell exosomes as miRNA drug delivery systems for the treatment of bladder cancer

**DOI:** 10.1002/cam4.4745

**Published:** 2022-04-20

**Authors:** Tianyao Liu, Tianhang Li, Yufeng Zheng, Xinyan Xu, Rui Sun, Shoubin Zhan, Xu Guo, Zihan Zhao, Wenjie Zhu, Baofu Feng, Fayun Wei, Ning Jiang, Jin Wang, Xi Chen, Feng Fang, Hongqian Guo, Rong Yang

**Affiliations:** ^1^ Department of Urology Affiliated Drum Tower Hospital, Medical School of Nanjing University Nanjing China; ^2^ Jiangsu Engineering Research Center for microRNA Biology and Biotechnology, State Key Laboratory of Pharmaceutical Biotechnology, School of Life Sciences Nanjing University Nanjing China; ^3^ Department of Urology Nanjing Drum Tower Hospital Clinical College of Nanjing Medical University Nanjing China; ^4^ Nanjing Drum Tower Hospital Clinical College of Jiangsu University Nanjing China; ^5^ Department of Pharmacology Nanjing Medical University Nanjing China

**Keywords:** adipose‐derived mesenchymal stem cells, bladder cancer, exosomes, miR‐138‐5p

## Abstract

**Objectives:**

Exosomes are essential mediators of intercellular communication as they transport proteins and RNAs between cells. Owing to their tumor‐targeting capacity, immune compatibility, low toxicity, and long half‐life, mesenchymal stem cell‐derived exosomes have great potential for the development of novel antitumor strategies. In this context, the role of exosomes produced by adipose‐derived mesenchymal stem cells (ADSCs) for the treatment of bladder cancer (BC) remains unclear. Here, we investigated the use of ADSCs as a source of therapeutic exosomes, as well as their efficacy in delivering the tumor suppressor miR‐138‐5p in BC.

**Methods:**

ADSCs stably expressing miR‐138‐5p were established using *Lentivirus* infection, and ADSC‐derived miR‐138‐5p exosomes (Exo‐miR‐138‐5p) were isolated from the cell culture medium. The effect of Exo‐miR‐138‐5p on BC cell migration, invasion, and proliferation was evaluated in vitro using wound healing, transwell invasion, and proliferation assays. The in vivo effect of Exo‐miR‐138‐5p was investigated using a subcutaneous xenograft mouse model.

**Results:**

Exo‐miR‐138‐5p prevented the migration, invasion, and proliferation of BC cells in vitro. Moreover, ADSC‐derived exosomes could penetrate tumor tissues and successfully deliver miR‐138‐5p to suppress the growth of xenograft tumors in vivo.

**Conclusions:**

The present results reveal that ADSC‐derived exosomes are an effective delivery vehicle for small molecule drugs in vivo, and exosome‐delivered miR‐138‐5p is a promising therapeutic agent for BC treatment.

## Graphical abstract

We found Exo‐miR‐138‐5p exerted anti‐migration, anti‐invasion and anti‐proliferation effects on the BC cells in vitro. Moreover, we observed that ADSCs‐exo could penetrate the tumor tissues and delivery RNA agents to suppress the growth of xenograft tumors in vivo.Our results not only reveal that ADSCs‐exo are an effective delivery vehicle for small molecule drugs in vivo but also confirm that ADSCs‐exo miR‐138‐5p is a promising therapeutic agent for BC treatment.

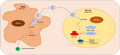

## 1 INTRODUCTION

Bladder cancer (BC) is one of the most common malignant tumors in the urological system. According to Bray et al., an estimated 549,393 new cases and 199,922 global deaths associated with BC were recorded in 2018.^1^ Only around 50%–60% of patients with advanced BC benefit from cisplatin‐based combination chemotherapy.^2^ In addition, side effects from traditional chemotherapy are very common, and many patients can only tolerate them over the short term. This means that there is an unmet need for new, safe, and effective treatments for advanced BC.

MicroRNA (miRNA), a type of single‐stranded RNA composed of 19–23 nucleotides, is known to promote selective mRNA degradation or translational inhibition via pairing with the 3′ untranslated region of the target gene mRNA.^3^, ^4^ Several studies have confirmed that dysregulation of miRNAs plays a crucial role in tumorigenesis and disease progression in various cancers.^5^, ^6^ Some miRNAs, including miR‐21, miR‐183, and miR‐20a, are upregulated in BC, where they contribute to various oncogenic properties.^7^, ^8^ Conversely, reports have described also the downregulation of several anti‐oncogenic miRNAs in BC, including miR‐101, miR‐125b, miR‐143, and miR‐145.^8^, ^9^ We previously demonstrated that miR‐138‐5p functioned as a tumor suppressor in BC by blocking proliferation, migration, and invasion in vitro, as well as tumor growth in vivo via its targeted inhibition of survivin.^10^ This suggests that miRNA‐based treatments may lead to novel therapeutic strategies for BC. The first miRNA drug for cancer entered clinical trials in 2013.^11^ However, synthetic RNA molecules are easy to hydrolyze and induce immune reactions during transport inside the body, which accelerates their clearance and limits their efficacy. Therefore, the development of a safe and effective delivery system for these therapeutics is critical to their success.

Exosomes are microvesicles enclosed by a lipid bilayer membrane. These vesicles are secreted by most cells and contain a variety of bioactive molecules, such as proteins, polysaccharides, lipids, metabolites, RNA, and DNA.^12^ Exosomes are transported via body fluids and absorbed by recipient cells, to which they deliver their bioactive molecules. Accordingly, exosomes are considered essential mediators of intercellular communication. In addition, exosomes exhibit no immunogenicity or biological toxicity, and can permeate blood vessels and biological barriers,^13^, ^14^ pointing to their potential as vectors for nucleic acid‐based drugs. Recent studies have shown that exosomes derived from mesenchymal stem cells (MSCs) possess higher tumor‐targeting capacity, better immune compatibility, lower toxicity, and longer half‐life than exosomes from other sources,^15^ making them perfect nano‐vehicles for delivering RNA agents in vivo. Previous studies have suggested that exosomes derived from both bone marrow mesenchymal stem cells (BMSCs) and umbilical cord mesenchymal stem cells (ucMSC) could be applied in targeted tumor therapy as they successfully packaged tumor suppressor miRNAs.^16^, ^17^ It remains unclear, instead, whether adipose‐derived mesenchymal stem cells (ADSCs), a different kind of adult stem cells derived from adipose tissues, could be used as sources of exosomes containing miRNA drugs, and whether they could effectively deliver their cargo to targeted BC cells.

This study aimed to investigate the feasibility of using engineered ADSCs as a source of therapeutic exosomes, and the efficacy of these exosomes in delivering miR‐138‐5p‐based therapeutics to BC cells and tumors in vitro and in vivo.

## 2 MATERIALS AND METHODS

### 2.1 Isolation and culture of ADSC

The detailed steps of ADSC separation are as follows:

First, the adipose tissue was removed from the para‐epididymis of mature rats and washed three times with PBS containing 100 μg/ml. Then, the adipose tissue was minced and digested with a mixture of 0.075% collagenase type I A in a 37°C water bath with vigorous shaking every 10 min until tissue complete digestion. Next, the cell suspension was filtered with a 70‐μm pore size cell sieve and then centrifuge for 5 min at 500 g. Finally, the isolated cells were evenly cultivated in DMEM/F12 medium containing 10% fetal bovine serum (FBS, Gibco), cultured in a humidified atmosphere at 37°C with 5% CO_2_.

### 2.2 ADSC characterization

Flow cytometry was utilized for identifying ADSC. First, the adherent ADSC cells were digested by trypsin and resuspended. Afterwards, cells were incubated using FITC–conjugated labeled monoclonal‐specific antibodies against CD45 (ab40763, abcam), CD29 (ab93758, abcam), CD31 (ab9498, abcam), and PE‐conjugated rat against CD90.1 (ab225, abcam) for 30 min. Finally, FACS Calibur cytometer was used for the analysis of ADSC‐specific surface marker expression.

### 2.3 ADSC‐derived exosome isolation

To harvest ADSC‐secreted exosomes, the ADSC (passage 4) were cultured with media of exosome‐free FBS. The exosome was isolated using ultracentrifugation method. Briefly, the supernatants were centrifuged at 500 *g* for 30 min, 10,000 g for 60 min to remove the cell debris and residues. The newly obtained supernatants were ultracentrifuged at 120,000 g for 90 min (Beckman Coulter Optima L‐80 XP) at 4°C. The exosome pellets adherent on the ultracentrifugation tube bottom were resuspended by PBS and stored at −80°C.

### 2.4 ADSC‐derived exosome characterization

The exosomes were observed by transmission electron microscopy (TEM, JEOL Ltd., JEM2010‐HT) to monitor their morphology. Nanoparticle tracking analysis (NTA) (NanoSight Technology) was used to evaluate the distribution, size, and quantity of ADSC‐derived exosome. The expression of specific surface markers of Calnexin (sc‐23,954, Santa Cruz), CD63 (sc‐5275, Santa Cruz), TSG101(sc‐7964, Santa Cruz), and CD9 (sc‐59,140, Santa Cruz) were evaluated using western blot analysis.

### 2.5 Cell culture and treatment

Bladder cancer cell lines T24 (highly invasive), 5637 (low invasiveness) were purchased from the Shanghai Institute of Cell Biology at the Chinese Academy of Sciences (Shanghai, China) with authenticated using short tandem repeat profiling, tested for mycoplasma contamination. T24 and 5637 were cultured in RPMI 1640 medium with 10% fetal bovine serum (FBS, Genial, South America Origin). All cells were incubated in a humidified incubator at 37°C with 5% CO_2_.

### 2.6 Cell transfection

For stable miR‐138‐5p overexpression in ADSC, *Lentivirus* expression system was utilized. In brief, *Lentivirus* combined with miR‐138‐5p (LV‐miR‐138‐5p) or negative control (LV‐vector) was constructed, and then ADSC were infected with LV‐miR‐138‐5p and LV‐vector. For miR‐138‐5p inhibition, the miR‐138‐5p inhibitor siRNA and negative control were transfected into ADSC with lipofectamine 3000 according to the manufacturer's instructions(Invitrogen). The RNA and proteins were harvested at 48 h after transfection to evaluate the transfection efficiency by RT‐qPCR and western blot.

### 2.7 Cellular uptake of ADSC‐derived exosome in vitro

For exosome uptake experiments, a PKH26 kit was used to label purified exosomes according to the manufacturers' instructions (PKH26GL‐1KT, Sigma‐Aldrich). Afterwards, the labeled exosomes were resuspended in PBS in the ultracentrifuge tubes and centrifuged at 120,000 g for 90 min. Then the exosome pellets were resuspended with PBS and cocultured with 5637 and T24 cell lines for 12 h with the dose of 0, 30, and 50 μg. After the coculture, the 5637 and T24 cells were fixed with 4% paraformaldehyde and stained with DAPI. Laser scanning confocal microscopy was used to detect the cellular uptake of ADSC‐derived exosome.

### 2.8 RT‐qPCR

Total RNA was extracted from cells and tissues by using Trizol Reagent (Invitrogen) according to the manufacturer's instructions. The harvested RNA of 1 μg was used to synthesize the complementary DNA by Prime Script RT MasterMix for RT‐PCR. To quantify the expression of miRNA, SYBR Green Premix Ex TaqTM kit (Takara) was utilized in Biosystems 7300 Sequence Detection System (Applied Biosystems). All the reactions were repeated in triplicate. Upon completion of PCR, the cycle threshold (CT) data were determined and the mean CT were derived from the triplicate PCRs. We selected U6 as the internal control for calculating the expression level of RNA. The relative expression of miRNA was determined as the Equation 2^−ΔΔCT^. The primers' sequences were follows: miR‐138‐5p (sense): GCGCGAGCGGGGGAAC; miR‐138‐5p (anti‐sense): GTCGTATCCAGTGCAGGGTCCGAGGTATTCGC.

ACTGGATACGACCGGCCT; U6 (sense): CTCGCTTCGGCAGCA.

CA; U6 (anti‐sense): AACGCTTCACGAATTTGCGT.

### 2.9 Protein extraction and western blot

The cells and tissues were treated with radio‐immunoprecipitation assay buffer (Beyotime), which was supplemented with phenylmethylsulfonyl fluoride (Beyotime). The supernatants were harvested after centrifugation and the protein concentration was detected by Pierce BCA protein assay kit (Thermo Scientific) following the manufacturer's instructions. Then the proteins were separated by 12.5% sodium dodecyl sulfate‐ polyacrylamide gel electrophoresis (Bio‐Rad). The expression of survivin protein was analyzed by western blot using survivin monoclonal antibody (71G4B7, 2808 s, Cell Signaling Technology). The protein levels were normalized by the same blots with a β‐actin antibody (Santa Cruz Biotechnology).

### 2.10 Wound healing assay

To evaluate the migratory ability of cancer cell under the treatment of Exo‐miR‐138‐5p, wound healing assay was performed. Briefly, T24 and 5637 cells were seeded in a 6‐well plates with the quantity of 5*10^5/well. When the density reached 60%, Exo‐miR‐138‐5p mimic and/or inhibitor and Exo‐vector was added into each well. PBS of the same volume was used as blank control. Twenty‐four hours after exosome treatment, a wound was made by the end of a 200‐μl pipette tip in each well. Photographs were taken at 0, 12, and 24 h to record the healing area of migratory cells and analyze the migratory ability of the cells.

### 2.11 Transwell assay

For transwell assay, we used a transwell chamber (Costar) precoated with Matrigel (BD Science) to detect the invasive ability of bladder cancer cell lines, including T24 and 5637. In brief, cancer cells suspended in FBS‐free media were seeded in the upper chambers (1* 10^5 cells/well). The lower chambers were added with 500 ul media with 10% FBS. After incubation of 24 h at 37°C, 5% CO2 in a humidified atmosphere, 4% paraformaldehyde, and stained with 0.5% crystal violet was used to fix the cells in the lower membrane, then a cotton swab was used to scrape the residual cells from the upper chamber. The numbers of invaded cells were calculated from randomly selected three fields of vision.

### 2.12 Animal experiments

Five‐weeks old male BALB/C nude mice were purchased from Model Animal Research Center of Nanjing University. The animal use protocol was reviewed and approved by the Animal Ethical and Welfare Committee (AEWC) (Approval No: IACUC‐2101006). The mice were injected with T24 cells (5*10^6 per mouse) subcutaneously into the right flank. When the minimal xenograft tumor volume reached 50mm^3^, the tumor‐loaded mice were randomly divided into two groups depending on the exosome delivery approach, one group used intratumoral injection method while the other group used tail vein injection method. In the intratumoral injection group, mice were further divided randomly into three subgroups and Exo‐vector (20 μg), Exo‐miR‐138‐5p (20 μg), and PBS (with equal volume of exosmoes) were respectively injected intratumorally. While in the tail vein injection group, mice were treated with Exo‐vector (100 μg), Exo‐miR‐138‐5p (100 μg), and PBS (with equal volume of exosmoes). The exosome was given seven times for every 4 days and the tumor volume was recorded every 4 days. The volume was determined as: volume = length × width^2^/2. One week after the last exosome treatment, the mice were all sacrificed and the tumor tissues were extracted, weighed, and prepared for further examination (Immunohistochemistry analysis, Western blot, and RT‐qPCR).

### 2.13 Hematoxylin and eosin and Immunohistochemistry analysis

The extracted tissues were first fixed with 4% paraformaldehyde fix solution, and then were embedded with paraffin. The paraffin sections were divided into two groups: one for immunohistochemistry (IHC) assay and the other for Hematoxylin and eosin(HE) staining. For IHC assay, xylene and alcohol were used to wash the paraffin sections for dewaxing, and then the dewaxed tissues were treated with citric acid antigen repair buffer (PH 6.0) for antigen repair. Subsequently, incubation of the sections was performed in 3% hydrogen peroxide solution for endogenous peroxidase blockade at RT for 25 min, and then the sections were washed by PBS. Bovine serum albumin (BSA) of 5% was used to block the sections for 30 min. After blocking, the sections were incubated with anti‐survivin antibody in a wet box at 4°C overnight. After incubation, the sections were washed with PBS (3*5 min, RT). The sections were then incubated with the second antibody for 50 min at RT. After the second‐ antibody incubation, the sections were washed again with PBS (3*5 min, RT). After second‐antibody incubation, coloration was performed with the diaminobenzidine chromogenic solution (Servicebio) (the positive color was brownish yellow). Next, the sections were rinsed with water to terminate the coloration. After that, hematoxylin was used to stain the nuclei and subsequently washed again with water. Finally, we dehydrated and sealed the sections and used the Olympus FSX100 microscope to detect the expression of protein.

### 2.14 Statistical analysis

All the results were repeated for at least three times in independent experiments. To compare the paired or unpaired data from two groups, student *t*‐test was used. All the data were shown in the form of mean ± SD. We selected one‐way analysis of variance (ANOVA) with Tukey's posthoc test to analyze the differences in the data among multiple groups. Data at multiple time points were analyzed using repeated‐measures ANOVA and Bonferroni posthoc test. Graphpad Prism statistical software (Version 5) and SPSS 25.0 statistical software package were used to perform these statistical analyses. *p* value <0.05 was considered as the standard for statistically significant difference.

## 3 RESULTS

### 3.1 Characterization of rat ADSCs

**FIGURE 1:**
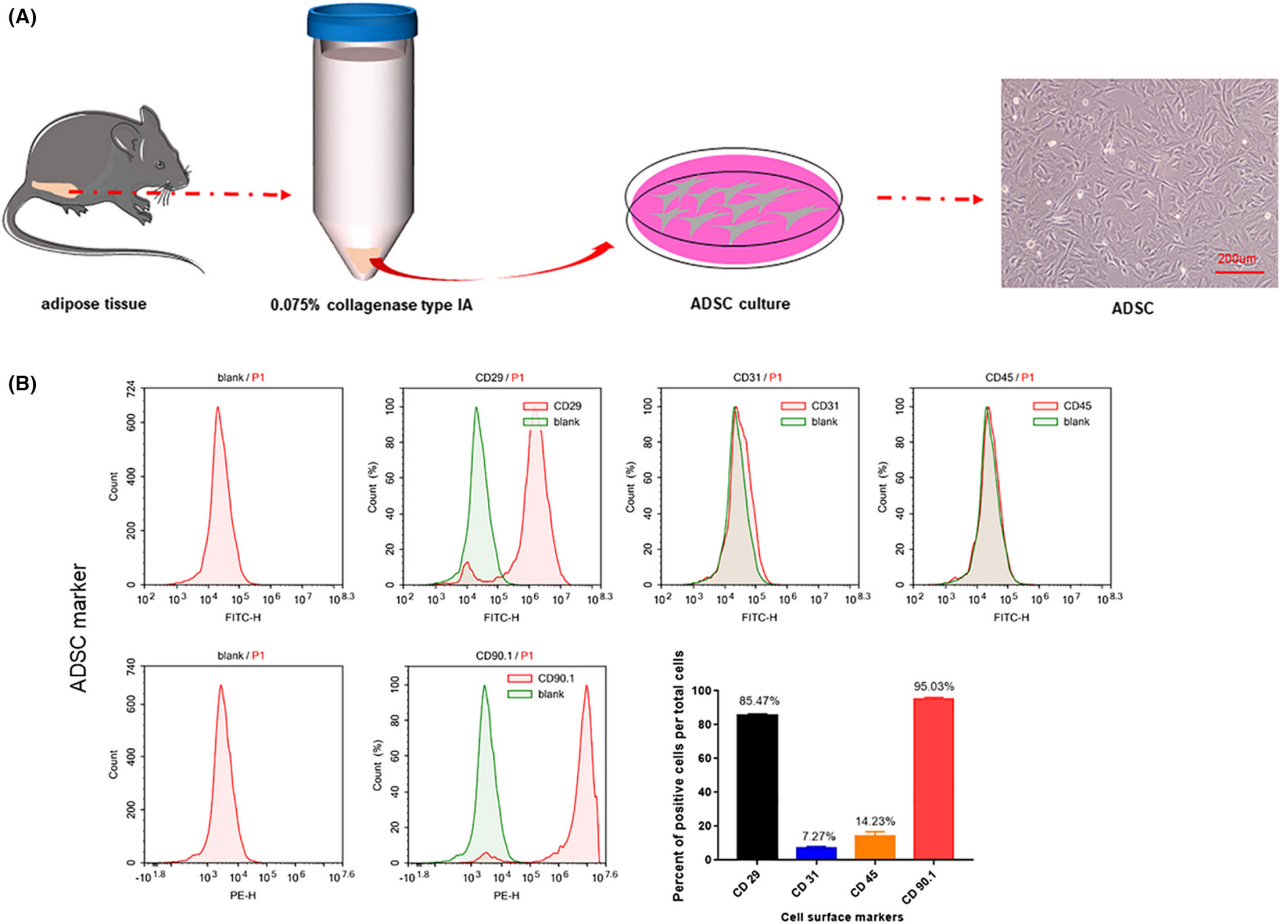
The characterization of rat ADSCs. (A) Isolation of adipose‐derived mesenchymal stem cells from rat paraepididymal adipose tissue. Primary ADSCs exhibit fibroblast‐like morphology. Scale bar, 200 μm. (B) ADSCs Surface Markers were detected by Flow Cytometry. Most of the mesenchymal stem cell markers CD29 and CD90.1 were strongly positive and only a few cells expressed endothelial cell‐related markers CD30, CD45

First, we isolated ADSCs from adult rat adipose tissue and confirmed their identity after expanding them in vitro. ADSCs were characterized by a fibroblast‐like morphology (Figure 1A). We evaluated the expression of stemness‐related surface markers in ADSCs at passage four using flow cytometry. Most MSC markers, including CD29 and CD90.1, were strongly positive; whereas only a minority of cells expressed endothelial cell‐related markers CD31 and CD45 (Figure 1B). These results suggest that we could accurately isolate ADSCs from adipose tissue and expand them in vitro.

### 3.2 Isolation and identification of ADSC‐derived exosomes

**FIGURE 2:**
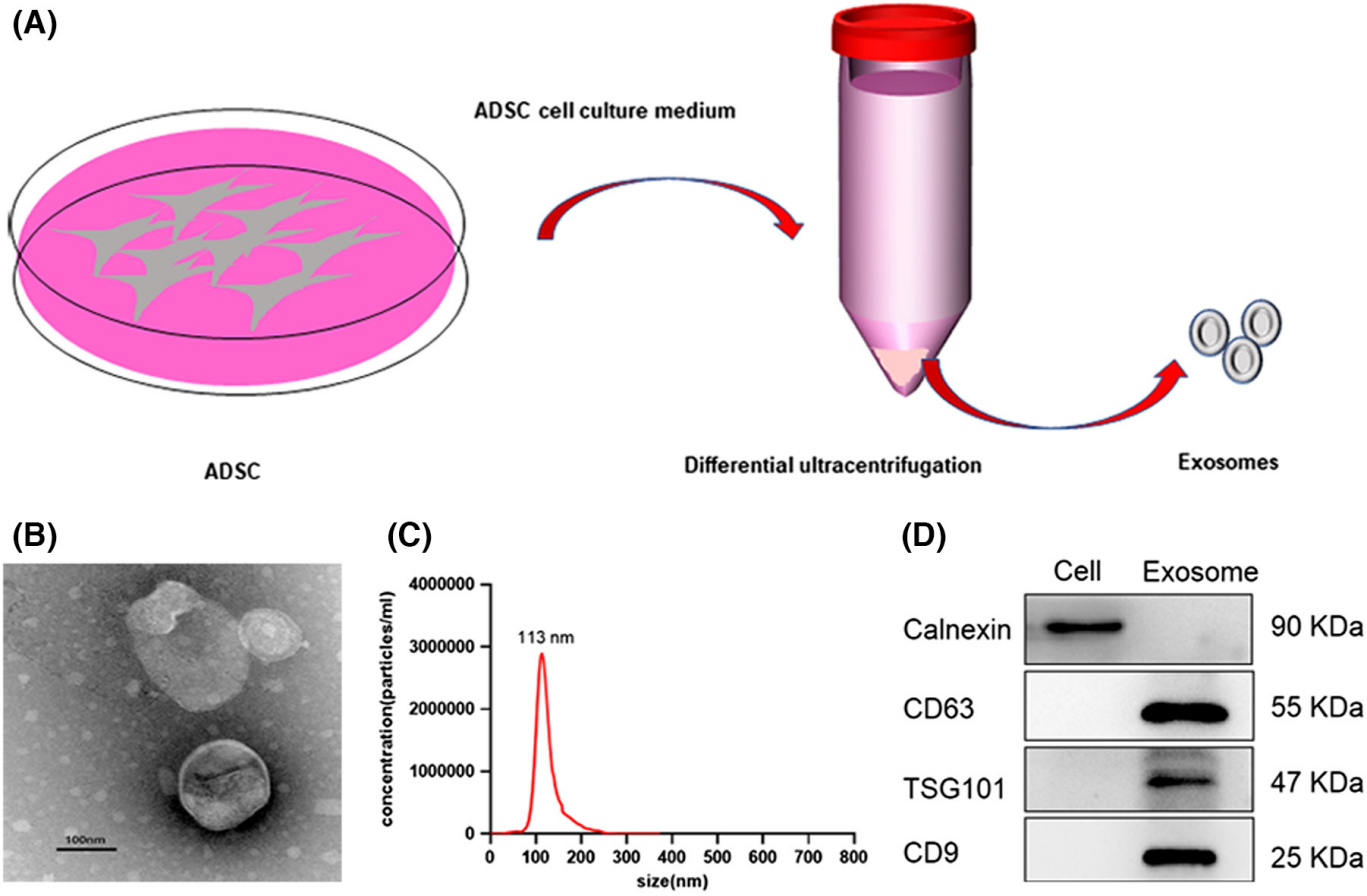
Isolation and identification of exosomes derived from ADSCs. (A) Isolation of exosomes from ADSCs by differential ultracentrifugation. (B) Morphology of exosomes observed by transmission electron microscopy (TEM), Scale bar, 100 nm. (C) Diameter distribution of exosomes detected by Nanosight analysis. (D) Exosome protein markers were detected by western blot

Exosomes were isolated via differential ultracentrifugation from ADSCs grown in medium containing exosome‐free serum (Figure 2A). Using transmission electron microscopy, we found that ADSCs produced lipid bilayer vesicles with the typical round or oval structure associated with exosomes. These structures had a diameter of about 100 nm and were hollow in the middle (Figure 2B). When we evaluated these particles by NanoSight, most vesicles appeared to have a diameter of 113 nm, although they ranged in size from 80 to 200 nm (Figure 2C). Western blotting revealed that CD9, CD63, and TSG101 were expressed on the surface of these vesicles but not on the corresponding cells, confirming their identity as ADSC‐derived exosomes (ADSCs‐exo) (Figure 2D). Concentration measurements indicated that we could isolate about 50 μg of exosomes from each 75 cm^2^ flask of ADSCs. Taken together, these results show that ADSCs can be used as exosome donors.

### 3.3 Engineered ADSCs secrete miR‐138‐5p‐enriched exosomes

**FIGURE 3:**
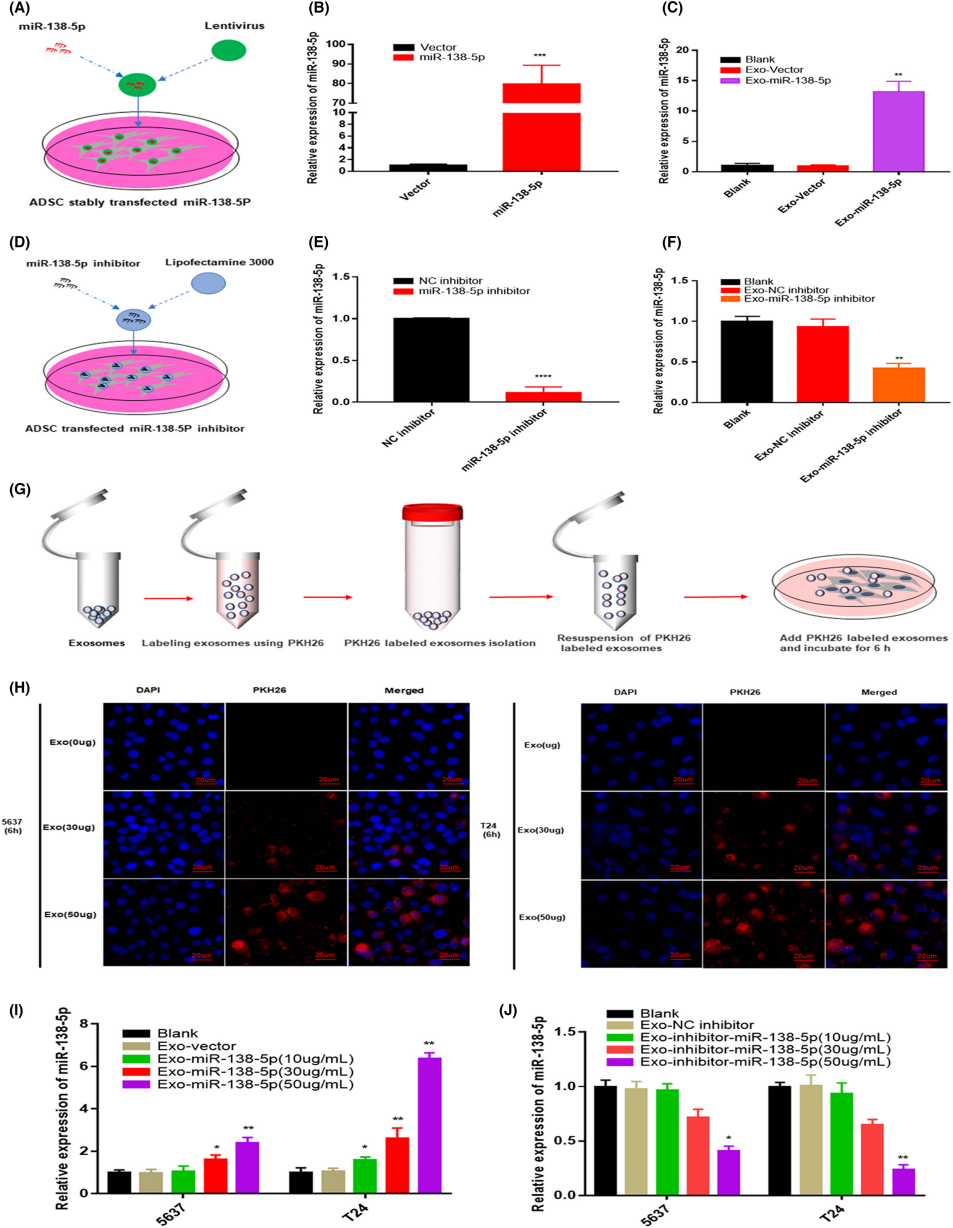
ADSCs could secrete exogenous miR‐138‐5p exosomes in a self‐assemble approach. (A) Construction of ADSCs stably expressing miR‐138‐5p with *Lentivirus*. (B) The relative expression level of miR‐138‐5p in ADSCs after stably transfected with miR‐138‐5p. U6 was used as internal reference. Data represent the mean ± SD. ***p < 0.001. (C) The relative expression level of miR‐138‐5p in exosomes after ADSCs stably overexpresses miR‐138‐5p. U6 was used as internal reference. Data represent the mean ± SD. **p < 0.01. (D) Transfection of miR‐138‐5p inhibitor into ADSCs by lipofectamine 3000. (E) The relative expression level of miR‐138‐5p in ADSCs after knockdown miR‐138‐5p. U6 was used as internal reference. Data represent the mean ± SD. ***p < 0.001. (F) The relative expression level of miR‐138‐5p in exosomes after ADSCs knockdown miR‐138‐5p. U6 was used as internal reference. Data represent the mean ± SD. **p < 0.01. (G) Schematic illustration shows that ADSC‐exo labeled with PKH26 and coculture with BC cells. (H) Exosomes internalization experiments showed that PKH26‐labeled exosomes (red) were taken up by BC cells. Nuclei were stained with DAPI. Scale bar, 20 μm. (I–J) The relative expression level of miR‐138‐5p in cocultured BC cells. U6 was used as internal reference. Data represent the mean ± SD. *p < 0.05, **p < 0.01

We used lentiviral transduction to generate a stable miR‐138‐5p‐overexpressing cell line capable of releasing miR‐138‐5p‐enriched exosomes (Figure 3A, Figure S1). The capacity of these ADSCs‐exo to deliver small molecule drugs was then investigated. Using RT‐qPCR, we confirmed that miR‐138‐5p expression was increased by more than 80 times in engineered ADSCs compared to control cells and by 13 times in ADSCs‐exo (Figure 3B, C). In contrast, when ADSCs were transfected with an Exo‐miR‐138‐5p inhibitor, we saw a significant decrease in miR‐138‐5p expression in both the engineered ADSCs and their exosomes compared to the control (Figure 3D–F). We then cultured 5637 and T24 BC cells with PKH26‐labeled ADSCs‐exo for 6 h and evaluated their uptake using confocal microscopy. The red fluorescent signal was observed in the cytoplasm and its intensity correlated positively with exosome concentration in both cell lines (Figure 3G, H). Next, we investigated the efficiency of ADSCs‐exo‐mediated transfer of miR‐138‐5p to BC cells. For this experiment, 5637 and T24 cells were cocultured with Exo‐miR‐138‐5p or an Exo‐miR‐138‐5p inhibitor at various concentrations (0, 30, and 50 μg/ml). The results showed that increasing the exosome concentration to 50 μg/ml increased the expression of miR‐138‐5p in 5637 and T24 cells by three and six times, respectively (Figure 3I, J). Therefore, we chose 50 μg/ml Exo‐miR‐138‐5p for all subsequent ex vivo experiments.

Overall, these results indicate that exogenous miR‐138‐5p can be loaded into ADSCs‐exo, likely by co‐opting the self‐assembly pathway, and that these constructs are fully transmitted to recipient cells.

### 3.4 Exo‐miR‐138‐5p suppresses BC cell migration, invasion, and proliferation in vitro

**FIGURE 4:**
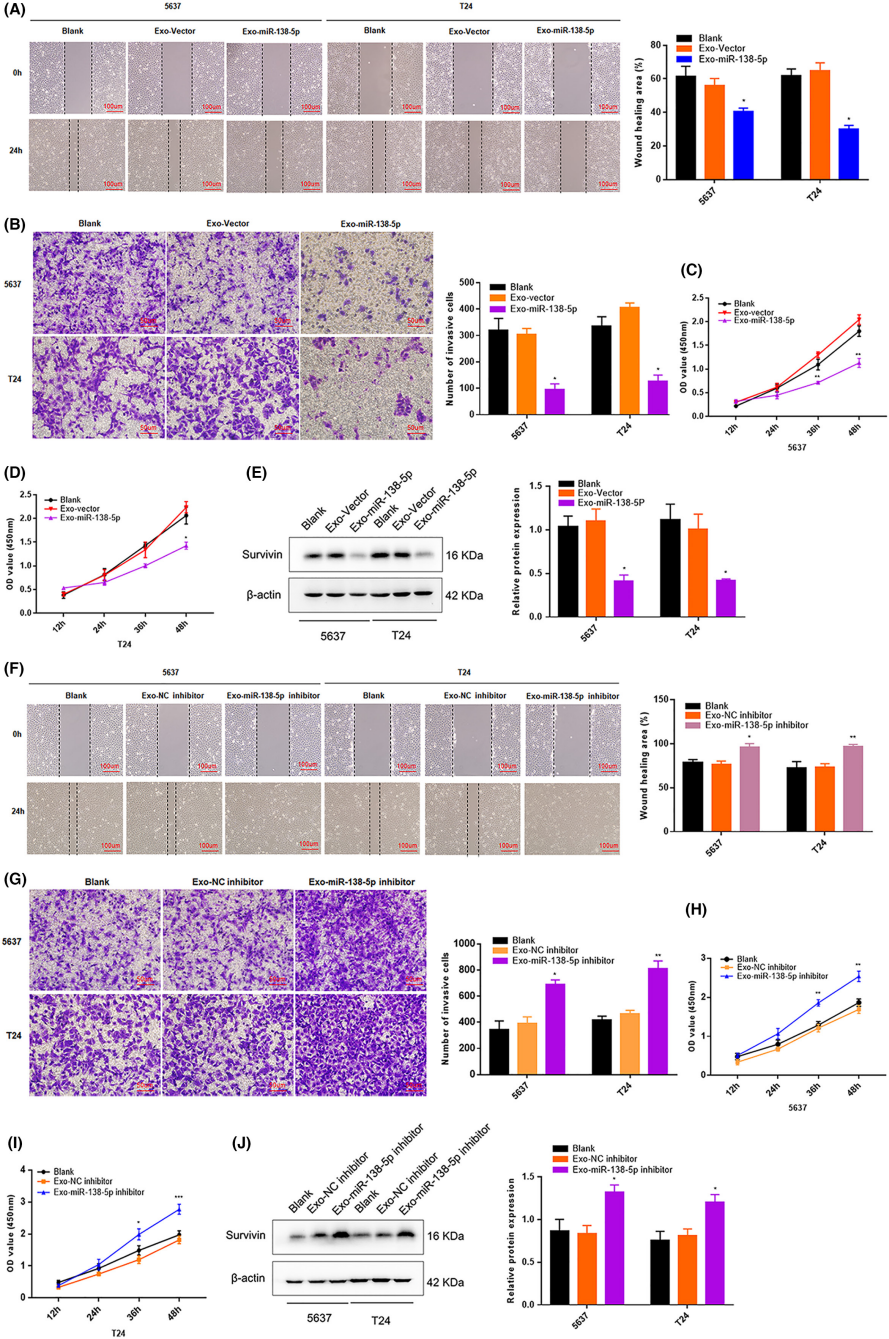
ADSCs‐derived exosomes miR‐138‐5p suppresses BC cells migration, invasion and proliferation in vitro. (A–D) The biological role of ADSCs‐exo miR‐138‐5p on cell migration, invasion, and proliferation capability were assessed by wound healing, transwell matrigel invasion assay and CCK‐8 assays in 5637 and T24. ADSCs‐exo miR‐138‐5p of 50 μg/ml was used as the experimental concentration. Wound healing assay, scale bar, 100 μm. Transwell invasion assay, scale bar, 50 μm. *p < 0.05, **p < 0.01. (E) After BC cells cocultured with ADSCs‐exo miR‐138‐5p, the expression level of survivin was determined by western blot analysis. β‐Actin used as internal reference. *p < 0.05. (F–I) After BC cells cocultured with ADSCs‐exo miR‐138‐5p inhibitor, the cells migration, invasion, and proliferation abilities were evaluated by wound healing assay, transwell matrigel invasion assay and CCK‐8 assays. ADSCs‐exo miR‐138‐5p of 50 μg/ml was used as the experimental concentration. Wound healing assay, scale bar, 100 μm. Transwell invasion assay, scale bar, 50 μm. *p < 0.05, **p < 0.01, ***p < 0.001. (J) The expression level of survivin was determined by western blot analysis. β‐Actin used as internal reference. *p < 0.05

Next, we cocultured 5637 and T24 cells with Exo‐miR‐138‐5p to determine whether ADSC‐derived Exo‐miR‐138‐5p suppressed BC progression in vitro. The biological function of treated cells was assessed using the wound healing, transwell invasion, and cell count kit‐8 assays. The migratory, invasive, and proliferative capabilities of both BC cell lines were notably attenuated following co‐culture with Exo‐miR‐138‐5p (Figure 4A–D). The expression of survivin, which is posttranscriptionally regulated by miR‐138‐5p, was also significantly reduced (Figure 4E). On the contrary, the migration, invasion, and proliferative capacities of both 5637 and T24 cells were increased following co‐culture with the Exo‐miR‐138‐5p inhibitor (Figure 4F–I), and the same pattern was observed with expression of survivin (Figure 4J). Collectively, these data show that Exo‐miR‐138‐5p exerted a tumor inhibitory effect in vitro.

### 3.5 Exo‐miR‐138‐5p inhibits bladder tumor growth in vivo

**FIGURE 5:**
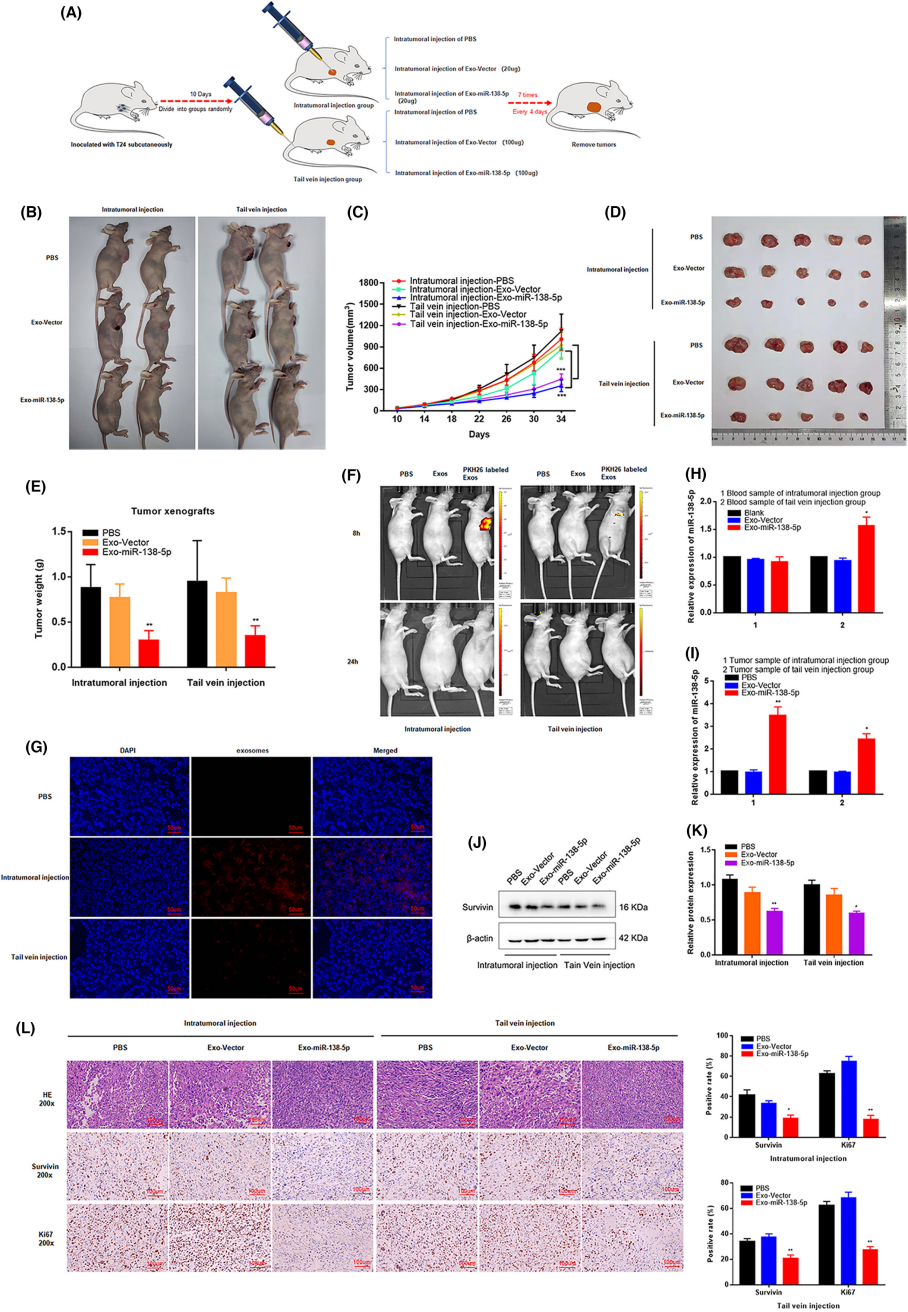
ADSCs‐derived exosomes miR‐138‐5p inhibits BC growth in vivo. (A) Schematic representation of the establishment of the xenograft model (1 × 107 cells per mice, *n* = 8 each group). (B–E) The growth rate and tumor weight significantly reduced in ADSCs‐exo miR‐138‐5p groups compared with the control groups. *p < 0.05, **p < 0.01, ***p < 0.001. (F) In vivo imaging system measured the delivery efficiency of exosomes in vivo. Fluorescence enrichment in tumor region after injection of PKH26‐labeled exosomes for 8 h. (G) Immunofluorescence was used to detect the distribution of exosomes in tumor tissues after injection of PKH26 labeled exosomes (red) 24 h. Scale bar, 50 μm. (H–I) The relative expression level of miR‐138‐5p in blood samples and tumor tissues of nude mice model. U6 was used as internal reference. Data represent the mean ± SD. *p < 0.05, **p < 0.01. (J, K) The expression level of survivin in tumor tissues was determined by western blot. β‐Actin used as internal reference. *p < 0.05. (L) Immunohistochemical representative images showed the survivin and ki67 positive cells in tumor tissue. Scale bar, 100 μm. *p < 0.05, **p < 0.01

Finally, we established a xenograft BC tumor model in BALB/c nude mice based on subcutaneous injection of T24 cells (Figure 5A) to evaluate the therapeutic potential of Exo‐miR‐138‐5p in vivo. After seven injections of Exo‐miR‐138‐5p or PBS, we noted that tumor growth was significantly reduced in the Exo‐miR‐138‐5p‐treated groups compared to the control (Figure 5B). Both tumor volume and weight were notably reduced after treatment with Exo‐miR‐138‐5p in the intratumoral and tail vein injection groups (Figure 5C–E, Figure S2). Based on fluorescently labeled Exo‐miR‐138‐5p and in vivo imaging, we demonstrated that these miR‐138‐5p constructs were transferred into tumor tissues by ADSCs‐exo. Fluorescent exosomes were significantly enriched in tumor tissues following 8 h of treatment with PKH26‐labeled exosomes, and the red fluorescent signal was observed in tumor tissues even 24 h post‐injection (Figure 5F, G). The miR‐138‐5p level was slightly elevated in blood samples from the tail vein treatment group when compared to the controls, but no difference was observed in the intratumoral injection groups (Figure 5H, I). Instead, miR‐138‐5p expression was elevated in the tumor tissue of both Exo‐miR‐138‐5p‐treated groups, while survivin protein expression was reduced (Figure 5J, K, Figure S3). These results reveal that miR‐138‐5p was successfully delivered to tumor sites using ADSCs‐exo.

HE staining of xenograft tumor tissues revealed significantly less cell mitosis in the tumor samples of Exo‐miR‐138‐5p‐treated groups, together with fewer survivin‐positive tumor cells. Likewise, the number of Ki67‐positive tumor cells were reduced in the Exo‐miR‐138‐5p‐treated groups compared with the control (Figure 5L). These results demonstrate that ADSCs‐exo can successfully deliver small molecule drugs aimed at treating BC in vivo.

## 4 DISCUSSION

As a class of essential regulatory molecules, miRNAs are involved in the occurrence and pathogenesis of various diseases, suggesting their untapped potential as therapeutic target genes and small molecule agents. Bouchie et al.^11^ and Li et al.^18^ demonstrated that exogenous antisense oligonucleotides or miRNA mimics could be delivered to specific tissues, where they exerted their corresponding biological function. Nevertheless, stable and effective delivery of exogenous miRNAs or antisense oligonucleotides to targeted organs remains a challenge. Previously, viruses, nanoparticles, and liposomes have been used as vectors for small RNA molecules and their transfer into tissues in vivo. However, these small RNA vectors have many disadvantages, including clearance by antibodies, macrophages, and extracellular complements, or stimulation of immune and toxic reactions within the cell. This means that a safe and effective drug delivery system for these small molecule drugs still needs to be developed to ensure the therapeutic success of these agents.

Exosomes are natural carriers secreted by most cells to transfer information, in the form of proteins or RNAs, between adjacent cells and distant organs. Exosomes have neither immunogenicity nor biological toxicity, and can permeate blood vessels and biological barriers.^13^, ^14^ In addition, exosomes have a longer half‐life than most artificial nanocarriers in vivo, leading to an increase in therapeutic time for their cargo.^19^

Exosome donor cells need to meet the following criteria: rapid amplification, increased exosome secretion, easy transfection, and high levels of biological safety. At present, most exosome donor cells are derived from HEK 293 T and dendritic cells (DC), but neither of these are suitable for clinical use, as the former exhibit strong tumorigenicity in vivo, while the latter are difficult to isolate and expand in vitro. MSCs are the only known human cells capable of naturally secreting large amounts of exosomes and exhibit specific tumor‐targeting properties.^20^, ^21^ MSC‐derived exosomes express the tumor tropism of their maternal cells, as well as CD47, which reduces immune clearance and prolongs their in vivo circulation time.^21^, ^22^ These characteristics render MSCs ideal sources of therapeutic exosomes and, consequently, delivery carries for exogenous RNA drugs.

Several studies have demonstrated that BMSC‐derived exosomes (BMSCs‐exo) and human umbilical cord mesenchymal stem cell (hucMSC)‐derived exosomes (hucMSCs‐exo) can be used as carriers for exogenous small molecule drugs in the treatment of various diseases.^23^, ^24^, ^25^ Naseri et al. found that BMSCs‐exo loaded with anti‐miR‐142‐3p regulated the expression of target genes APC and P2X7R, thus inhibiting the progression of breast cancer.^26^ Chen et al. reported the therapeutic potential of BMSCs‐exo miRNA‐150‐5p in rheumatoid arthritis, whereby they MMP14 and VEGF expression.^27^ Wei et al. reported a novel therapeutic strategy for myocardial ischemia and reperfusion injury using miRNA‐181a delivered by MSCs‐exo.^28^ Cai and colleagues showed that BMSCs‐exo loaded with miR‐9‐3p regulated the expression of endothelial cell‐specific molecule 1 (ESM1) in BC cells, thereby inhibiting disease progression.^17^ Similarly, Jia et al. demonstrated that hucMSCs‐exo could deliver exogenous molecules in vivo, and that exosome‐mediated delivery of miR‐139‐5p could suppress BC progression via inhibition of the ring finger protein polycomb repressor complex 1.^16^

ADSCs are often more readily available than other MSCs and are easily expanded in vitro. Moreover, they are also highly stable and exhibit low levels of immunogenicity in vivo.^29^, ^30^ Chen et al. showed that ADSCs‐exo miR‐375 could facilitate bone regeneration in a rat model.^31^ A similar study demonstrated that GDNF‐modified ADSCs released GDNF‐rich exosomes, which could be transferred to renal fibrosis tissues and ameliorate peritubular capillary loss via the activation of the SIRT1/eNOS signaling pathway.^32^ In contrast, the function of ADSCs‐exo in BC has remained vastly unknown. In this study, we demonstrate that ADSCs were easily transfected by *Lentivirus* and successfully secreted exosomes containing therapeutic agents. Furthermore, exogenous ADSCs‐exo miR‐138‐5p could be internalized by BC cells and exert the same antitumor effect as an miR‐138‐5p mimic transfected by liposomes in vitro.

The tumor suppressor gene miR‐138‐5p plays an important regulatory role in the tumorigenesis of various cancers. He et al. reported that miR‐138‐5p was involved in regulating angiogenesis in glioma via its modulation of SOX13 expression.^33^ Bai et al.^34^ and Yang et al.^35^ revealed that miR‐138‐5p suppressed growth and metastasis of lung cancer and hepatocellular carcinoma by targeting FOXC1. Moreover, miR‐138‐5p plays a tumor‐suppressing function in glioblastoma multiforme and colorectal cancer by inhibiting the expression of NFAT5 and ZEB2, respectively.^36^, ^37^ We previously demonstrated that miR‐138‐5p was downregulated in BC cell lines and tissues, which promoted tumor development.^10^ In addition, the expression level of miR‐138‐5p is closely related to the pathological stage, grade, and overall survival time of BC from multiple databases (Figure S4). As such, miR‐138‐5p is of strategic importance for the development of various tumor‐targeting therapies.

Numerous studies have shown that drugs delivered by exosomes can effectively suppress tumor progression in vivo via intratumoral administration. However, few studies have compared the therapeutic effects of intratumoral and intravenous administration in the same tumor. Herein, we tried two exosome injection strategies for BC xenograft models. We found that intratumoral and caudal vein administration exerted significant antitumor effects, but this effect was enhanced with the intratumoral route. This may be caused by local enrichment of exosome‐carried drugs following such targeted injection. Next, we explored the delivery effect of ADSCs‐exo in a xenograft tumor model by tracking the distribution of fluorescently labeled exosomes in vivo. We observed that ADSCs‐exo could penetrate the tumor tissues and confirmed that intratumoral administration facilitated exosome delivery compared to tail vein injection. This was also consistent with the antitumor effects reported for each of these two administration methods. Based on these findings, we hypothesized that increasing the dose of exo‐agent might compensate for the loss of transportation in vivo and increase drug delivery to tumor tissues. Furthermore, loading a specific BC‐targeting sequence on exosome membranes might cause enrichment of such exosomes in the blood. This hypothesis will be verified in future experiments.

In summary, exosomes are widely accepted as effective delivery vehicles for a variety of small molecule drugs, but some limitations need to be addressed before exosome delivery vectors can be used in clinical practice. These include a lack of simple, standardized methods for their isolation and purification, and their inconsistent secretion efficiency when using engineered MSCs.

## 5 CONCLUSIONS

Taken together, our results confirm that ADSCs‐exo could penetrate BC tissue and deliver miRNA‐based drugs to tumor cells in vivo. In addition, we compared intratumoral and caudal vein administration in the treatment of BC and found that both methods could significantly inhibit tumor growth. These results suggest that ADSCs‐exo represent an effective delivery vehicle for small molecule drugs in vivo and that Exo‐miR‐138‐5p is a promising therapeutic candidate for BC treatment.

## CONFLICT OF INTEREST

The authors declare that there are no conflict of interest.

## AUTHOR CONTRIBUTIONS

RY, FF, and HQG: Conception and design, financial support. TYL, THL, and YFZ: Data analysis and interpretation, collection and assembly of data and manuscript writing. XYY, RS, SBZ, XG, and WJZ collection and assembly of data. ZHZ, BFF, FYW, and NJ: Collection of data. JW and XC: Data analysis.

## ETHICS APPROVAL AND CONSENT TO PARTICIPATE

The animal use protocol was reviewed and approved by the Nanjing University Animal Ethical and Welfare Committee (Approval No: IACUC‐2101006).

## Supporting information


Figure S1

Figure S2

Figure S3

Figure S4

Figure S5

## Data Availability

All data generated or analyzed during this study are included in this published article.
