# Supplementary figures and images for: Evaluating adipose‐derived stem cell exosomes as miRNA drug delivery systems for the treatment of bladder cancer
Source: Cancer Med. 2022 Apr 20;11(19):3687–99. doi: 10.1002/cam4.4745 (PMC9554444; doi:10.1002/cam4.4745)

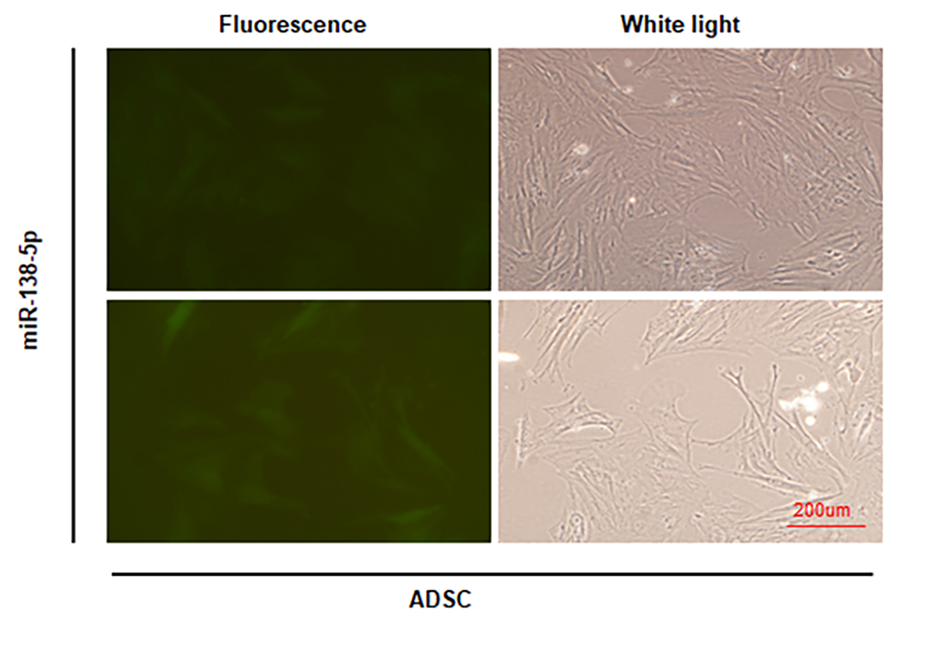

Supplement: Supplementary file 1 — Figure S1 [file CAM4-11-3687-s002.tif]

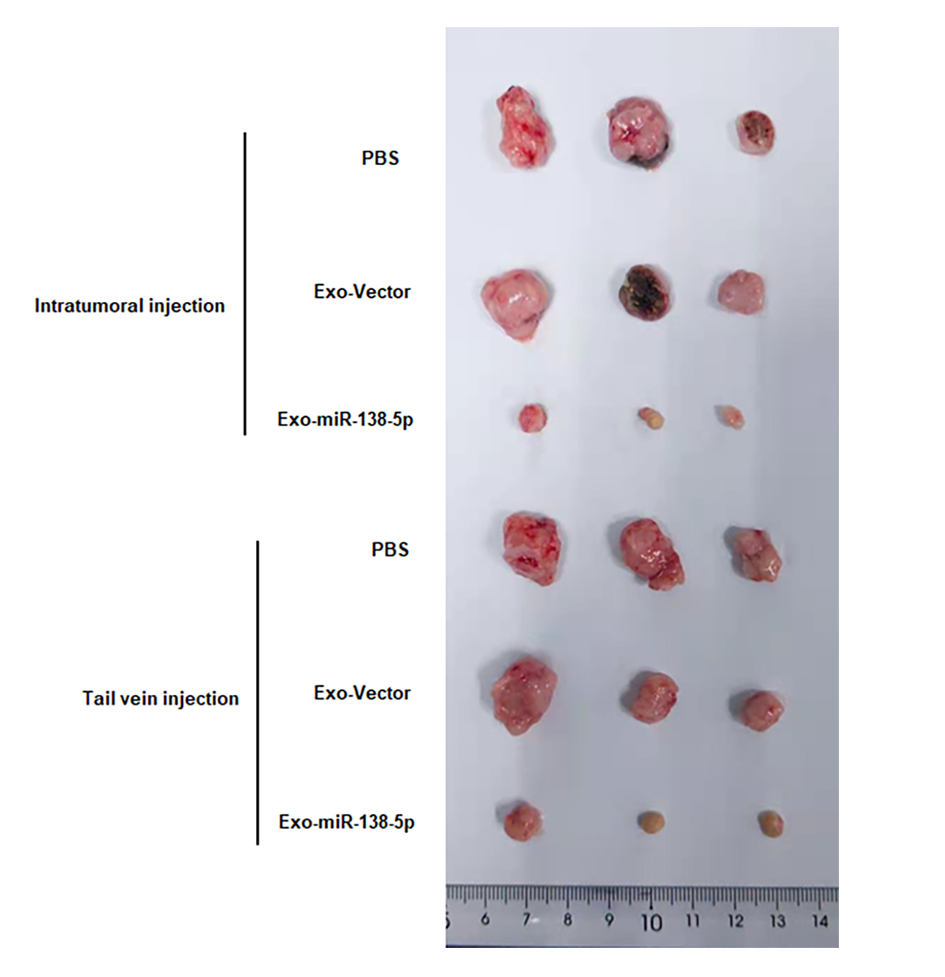

Supplement: Supplementary file 2 — Figure S2 [file CAM4-11-3687-s001.tif]

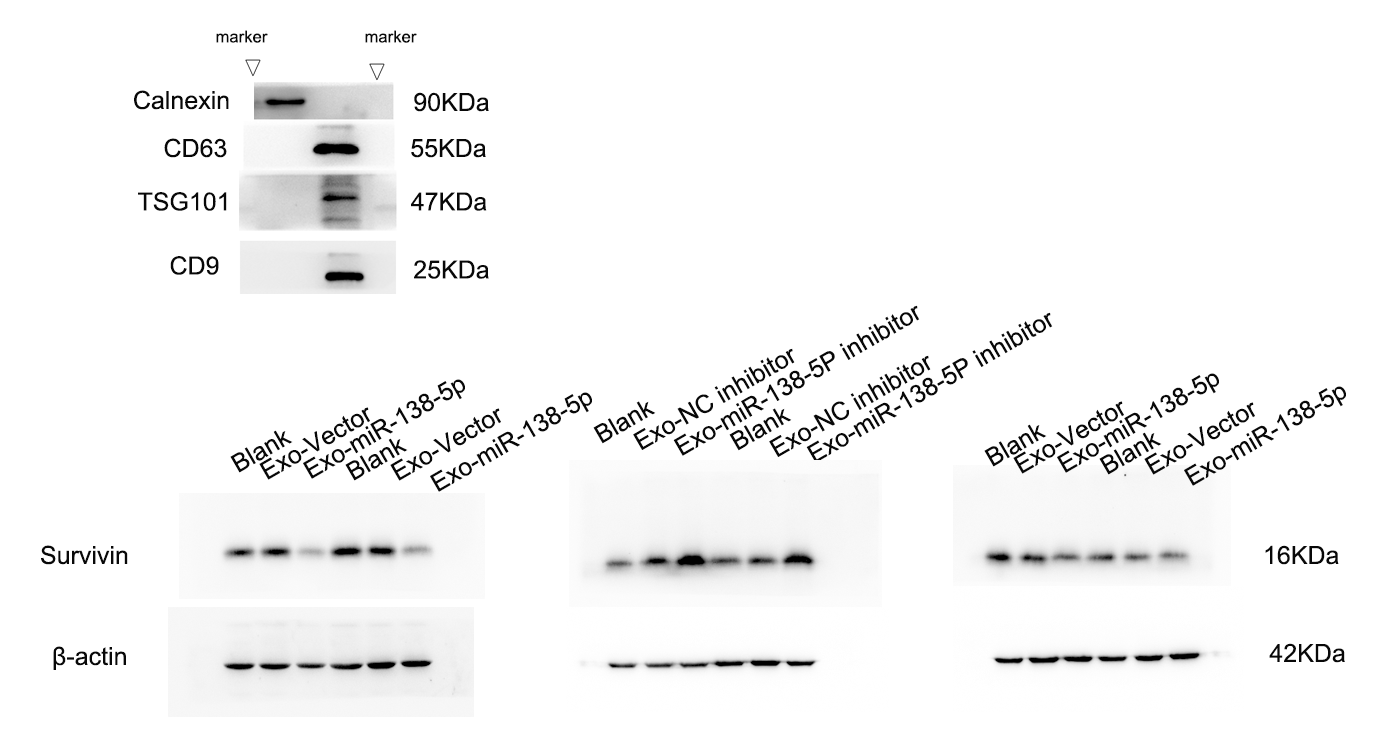

Supplement: Supplementary file 3 — Figure S3 [file CAM4-11-3687-s004.tif]

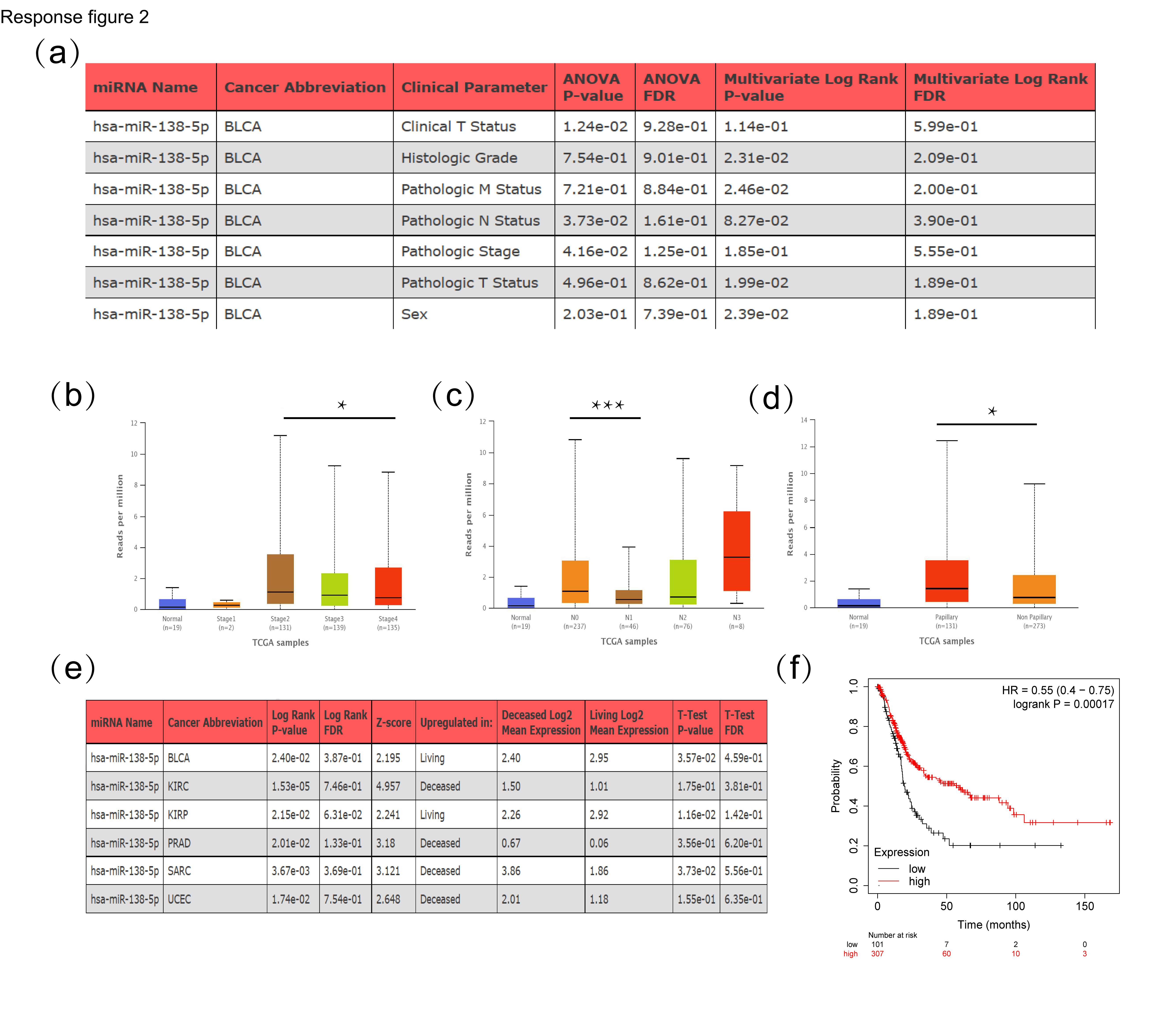

Supplement: Supplementary file 4 — Figure S4 [file CAM4-11-3687-s005.jpg]

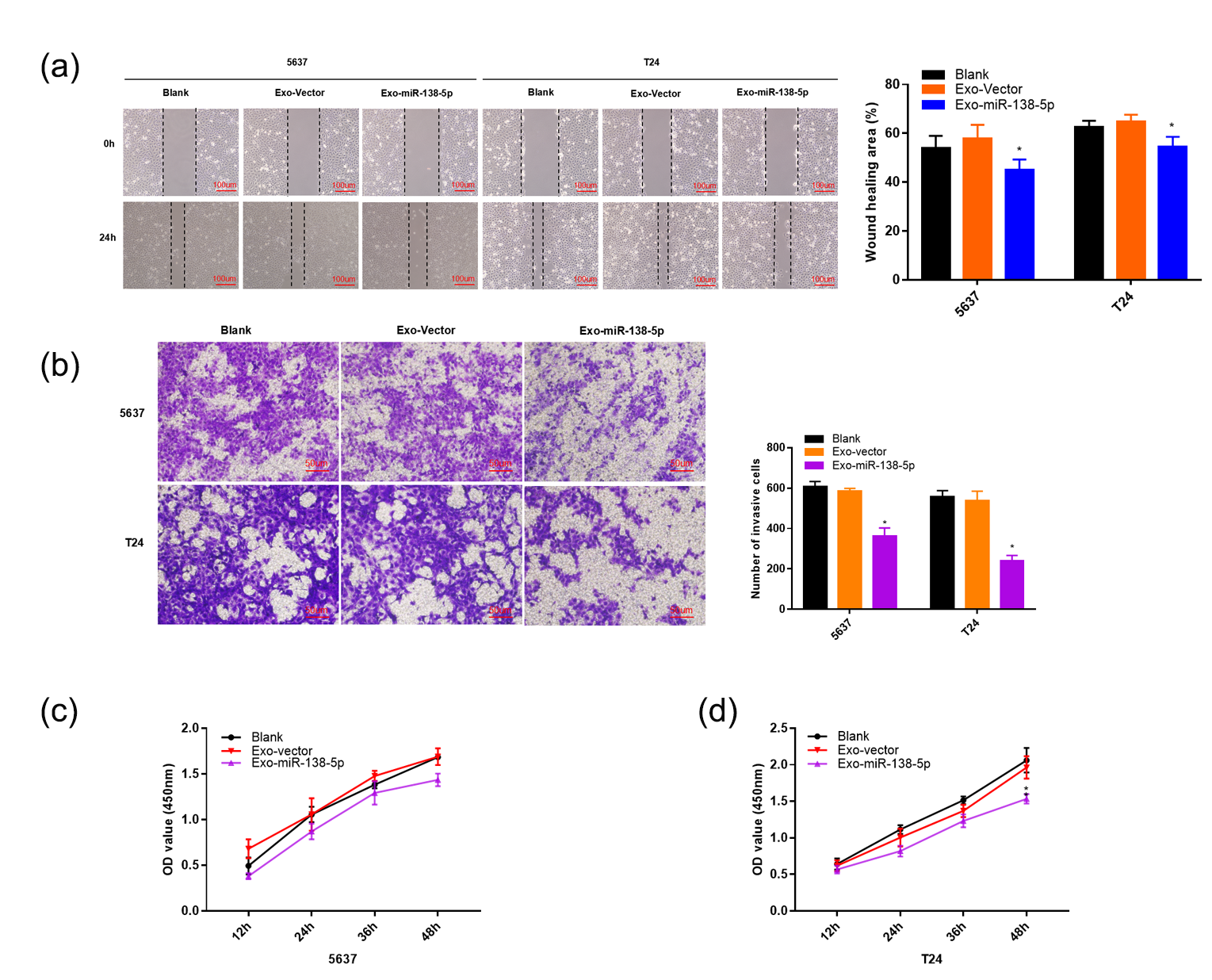

Supplement: Supplementary file 5 — Figure S5 [file CAM4-11-3687-s003.tif]
